# Experimental investigation on slotted Koch snowflake fractal patch rectenna

**DOI:** 10.1038/s41598-025-18152-1

**Published:** 2025-10-03

**Authors:** Ming Wei Seng, Hussain Lazzan Shareef, Ping Yi Chan, Yu Heng Teh, Calvin Fu Yuan Low

**Affiliations:** 1 Department of Electrical and Robotics Engineering, School of Engineering, Monash University Malaysia, Jalan Lagoon Selatan, Bandar Sunway, 47500 Subang Jaya, Selangor Malaysia; 2 Centre for Net-Zero Technology, Monash University Malaysia, Jalan Lagoon Selatan, 47500 Bandar Sunway, Selangor Malaysia; 3 Medical Engineering and Technology Hub, School of Engineering, Monash University Malaysia, Jalan Lagoon Selatan, 47500 Bandar Sunway, Selangor Malaysia

**Keywords:** Devices for energy harvesting, Electrical and electronic engineering

## Abstract

Radio frequency energy harvesting, a sustainable power source for IoT devices, faces limitations due to its low power density. However, existing slotted fractal antenna designs often lack systematic optimization and do not emphasize the 2 GHz band, which is crucial for urban RF energy harvesting. This article proposes a rectenna design featuring a Koch snowflake fractal patch antenna with optimized rectangular slots to enhance performance. A comparative study of single-slot and double-slot configurations revealed that double slots improve return loss peak depth and introduce additional resonance modes. The antenna was optimized using a multivariate parametric modelling-based approach with particle swarm optimization for the 2 GHz band. The 102 mm × 102 mm fabricated antenna exhibited − 26.8 dB return loss at 2.07 GHz, validated by experiments. Hence, the work demonstrated the feasibility of incorporating rectangular slots onto the Koch snowflake fractal patch. Furthermore, a half-wave rectifier circuit with a Pi matching network was fabricated. Initial discrepancies between the fabricated and simulated rectifier circuit resonant frequencies were observed. To address this, a microstrip line was introduced for impedance matching, and a capacitor was replaced with that of a higher operating frequency to minimize the parasitic effect due to the self-resonant frequency, thereby improving RF power transfer. The improved fabricated rectifier achieved over 20% RF-DC efficiency from an input power range of − 16 to 16 dBm at 1.88 GHz. Future work will focus on antenna size optimization and refined rectifier circuit simulations to develop a fully functional rectenna system for efficient energy harvesting.

## Introduction

With the exponential growth of the Internet of Things (IoT), the demand for wireless sensors within IoT systems has surged. However, most of these wireless devices rely on batteries, posing sustainability challenges due to their finite lifespan^1^. This leads to a substantial daily increase in discarded batteries worldwide, contributing to environmental pollution when the batteries are not disposed of properly. In light of these concerns, radio frequency (RF) energy harvesting has emerged as an alternative power source for IoT devices, particularly with the advancement of low-power wireless technologies^1^. Through the rapid advancement of wireless technologies, the ambient RF energy, generated by sources like cellular base stations, Wi-Fi routers, and electronic devices, has become increasingly prevalent. Rectennas, short for rectifying antenna, enable the conversion of ambient RF energy into direct current electrical power. A rectenna system comprises key components, including a receiving antenna, an impedance-matching network, and a rectifier circuit, working together to harvest energy from surrounding RF waves. Despite its advantages of unlimited availability and independence from factors like time and weather, RF energy harvesting faces the challenge of low power density^1,2^. This limitation has hindered the commercialization of rectennas, despite research efforts dating back to the early 1970s.

**Fig. 1:**
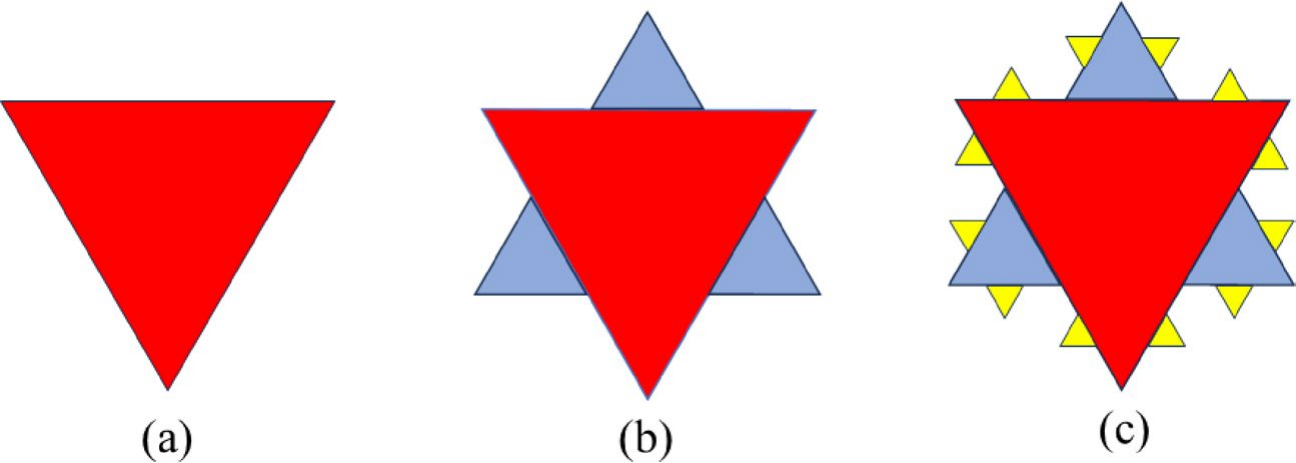
Koch snowflake geometry till 2nd iteration. (**a**) 0th iteration. (**b**) 1st iteration. (**c**) 2nd iteration.

Researchers continuously explore antenna, impedance-matching network, and rectifier circuit designs to enhance rectenna system efficiency. In antenna design, investigations focus on characteristics like substrate selection, patch dimensions, and shape to optimize antenna performance, quantified through metrics like resonance frequency, return loss, and bandwidth^3^. Miniaturizing antennas with minimal performance trade-offs presents a persistent challenge. Various strategies have been proposed to address this challenge, including employing high dielectric constant materials, incorporating slots, slits, shorting posts, and employing meandering slits on patch antennas^2^. For example, Jameel et al.^4^ demonstrate the feasibility of an ultra-wideband antenna using a slotted patch resonator with a Locked-Key topology. Fractal geometry has gained popularity for achieving size reduction and multiband functionality^5^. Unique fractal patterns like Sierpinski gaskets, Koch snowflakes (refer to Fig. 1), and Hilbert curves inspire researchers in their antenna designs^5,6^.

Through reviewing previous studies^7^, it is found that slots were incorporated in patch antennas, but the slotted fractal designs rarely target frequency at 2 GHz band, which is crucial for harvesting energy from urban RF environments. For instance, Dahiya et al.^8^ designed a slotted fractal antenna for bandwidth enhancement but focused on satellite communications (X-band (8–12 GHz) and Ku-band (12–18 GHz)), neglecting frequencies like WiFi (2.4/5 GHz) or 5G sub-6 GHz bands that dominate urban RF environments. Similarly, Ez-Zaki et al.^9^ integrated slots onto the ground plane of a fractal antenna to improve matching conditions. Their design targeted 5.9 GHz (for sub 6 GHz Vehicle-to-Everything Communication) without addressing rectenna efficiency or energy harvesting potential in the 2 GHz range.

While various studies have explored the integration of slots within fractal antennas in general (may not be for energy harvesting) utilizing different slot shapes and geometries, many lack systematic optimization. For instance, the study by Dahiya et al.^8^ incorporates fractal slot geometry to enhance gain and impedance bandwidth. The work focuses on the design to improve the performance and decrease effective area with the rectangular ring slotted and circular slotted fractal. However, the optimization of slot parameters is not thoroughly discussed. Similarly, another paper by Ez-Zaki et al.^9^ explores slot integration to reduce mutual coupling and improve isolation by incorporating double negative metamaterial structures on the substrate. Though slots are employed to improve impedance bandwidth, their optimization is not a primary focus in this work. Additionally, Fakharian^10^ introduces a thin rectangular slot to optimize resonance frequencies and bandwidth, but the study lacks detailed slot optimization processes. Although Parikh and Singh^11^ demonstrated slot placement impacts resonance, their work did not address rectenna efficiency or practical deployment barriers.

Compounding this, existing studies on slotted fractal designs often prioritize higher-order fractal iterations or intricate slot geometries, complicating fabrication and limiting scalability for low-cost IoT applications. The absence of a holistic design framework results in trade-offs between miniaturization, manufacturability, and efficiency—barriers that hinder real-world implementation.

To address these gaps, this study introduces a first-iteration Koch snowflake fractal antenna with a double-rectangular slot configuration, systematically optimized for ambient RF harvesting. The choice of a first-iteration fractal enhances manufacturability using standard PCB fabrication techniques, ensuring practical scalability. The double-rectangular slot placement is strategically optimized to enhance return loss and introduce a new resonance frequency, addressing key efficiency limitations. This optimization is achieved using particle swarm optimization (PSO), targeting ambient RF energy hotspots (2 GHz band) to maximize harvesting potential. This combination of features represents a novel approach to improving rectenna efficiency through an optimized fractal-slot synergy. The proposed design achieves miniaturization, multiband functionality, and enhanced impedance matching.

We have also designed and fabricated a rectifier circuit and an impedance-matching network using Advanced Design System (ADS) of version 2020 (https://www.keysight.com/my/en/lib/software-detail/computer-software/pathwave-advanced-design-system-ads-software-2212036.html). Simulations and experiments are conducted to validate the proposed antenna and rectifier circuit. The results demonstrate that the antenna exhibits a good overall return loss and multiband functionality, while the rectifier circuit shows a slight discrepancy but achieves 20% RF-DC efficiency at 1.88 GHz across an input power range of -16 dBm to 16 dBm.

## Description of the Koch snowflake fractal patch antenna

The starting point for this study is the Koch snowflake fractal patch antenna. The Koch snowflake geometry is generated through an iterative process, starting from a basic equilateral triangle (0th iteration), as shown in Fig. 1a in red. In the first iteration, each side of the triangle is divided into three equal segments, and an outward-pointing equilateral triangle is added to the middle segment of each side. This results in a six-pointed star shape, seen in Fig. 1b, where the added blue triangles represent the first iteration. The rule of varying the geometry follows a recursive process in each iteration, every straight-line segment is divided into three equal parts, and the middle segment is replaced with two segments that form an outward-pointing equilateral triangle. This transforms each straight segment into a four-segment structure, increasing the perimeter and complexity with each iteration. In the second iteration, the same process is recursively applied to each line segment, adding even smaller triangles, which are highlighted in yellow in Fig. 1c. Each iteration increases the perimeter and complexity of the structure while reducing the enclosed area.

**Fig. 2:**
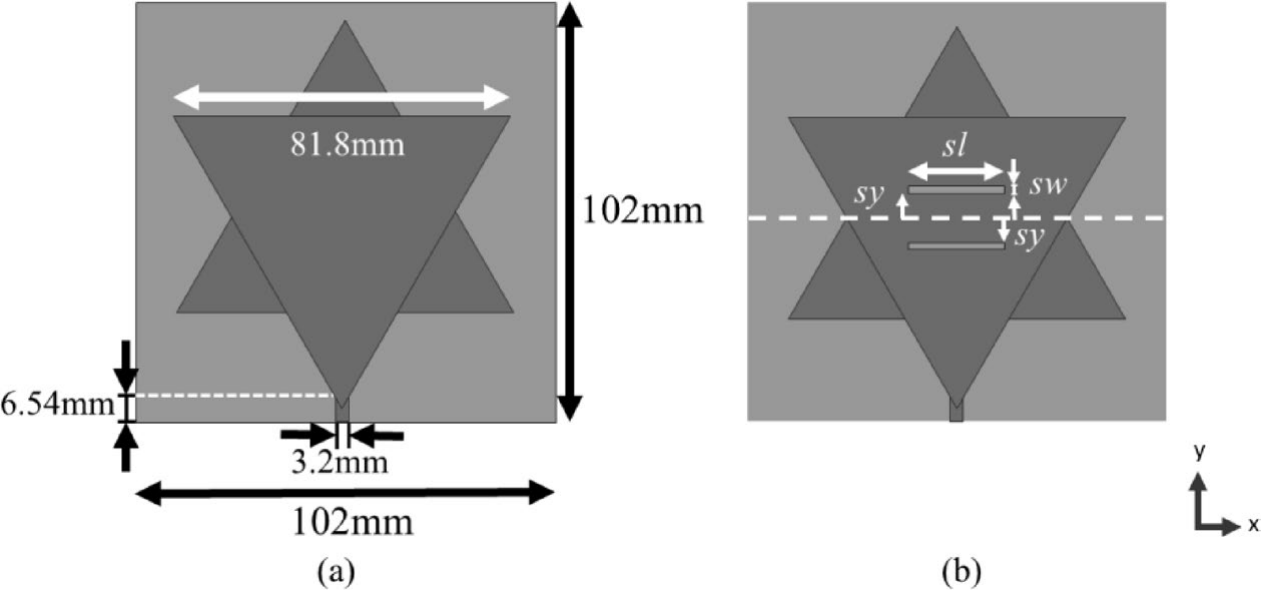
(**a**) Dimension of the initial Koch snowflake fractal patch antenna design. (**b**) Visual representation of the optimization key input parameters defined.

Specifically, a Koch snowflake design with a first-iteration fractal pattern, including slots, has been implemented. The decision to use just the first iteration is driven by practical considerations related to standard PCB manufacturing techniques. Subsequent iterations of the Koch fractal introduce increased complexity. By restricting the analysis to the first iteration, it ensures that the design remains feasible and cost-effective for practical implementation using widely available and simple manufacturing techniques. This approach balances performance optimization with manufacturability, making the design more suitable for real-world applications. The initial phase of the study involves determining the appropriate substrate size and the dimensions of the Koch snowflake fractal patch. In order to achieve this, it is required to gather data and conduct research to identify the optimal frequency range for tuning the rectenna design. Given the pervasive use of wireless communication across inhabited areas, there has been a corresponding rise in ambient radio frequencies available for energy harvesting. A literature review indicates that many rectennas are tuned within the 2 GHz range^12,13^. This frequency choice is primarily attributed to the minimal attenuation experienced by signals in this range within the atmosphere of the Earth^12^. Additionally, spectrogram analyzes of urban and semi-urban locations reveal distinct signal intensity peaks within this range^13^. In order to proceed, preliminary simulations have been carried out utilizing the Momentum tool in Advanced Design System (ADS) software. This initial simulation step serves to ascertain the dimensions of the patch and substrate that allow for optimal tuning within the 2 GHz frequency range. The patch and substrate dimensions can be found in Fig. 2a.

The subsequent phase of this study focuses on integrating slots into the antenna design to enhance the peak depth of return loss. Initially, a comparative analysis between single rectangular slot and double rectangular slots configurations was conducted. The results can be found in the Result and Discussion section, demonstrate that the double rectangular slots design outperforms the single rectangular slot configuration, introducing additional resonance modes and achieving a greater peak depth in return loss at the target frequency in the 2 GHz range. As a result, this study adopts the implementation of double rectangular slots. The positioning and dimensions of these slots are further refined using a multivariate parametric modeling-based approach, specifically employing particle swarm optimization (PSO) techniques. The optimization process focuses on key input parameters, which are outlined in Fig. 2b. In particular, “*sw*” signifies the width of the slot, “*sl*” represents the length of the slot, and “*sy*” indicates the vertical distance of the upper and lower slots above and below the center of the Koch snowflake, respectively. Figure 2b provides a visual representation of these definitions and measurements.

With the input parameters established, the subsequent step involves the selection of the output parameters, which directly correspond to the performance of the antenna. By incorporating both the input and output parameters, a mathematical model can be developed to establish a relationship between the input and output parameters. For a more systematic integration with the PSO method, output parameters must be quantifiable numerically, allowing for straightforward calculations and retrieval through simulation techniques. The selected output parameters hold fundamental significance in evaluating antenna performance, making them pivotal for analysis. The chosen output parameters are resonant frequency (*RF*), bandwidth (*BW*), and peak depth (*PD*).

In order to conduct a comprehensive exploration of the output parameter space, a series of multiple parametric sweep simulations is executed utilizing ADS. Simulation sets are performed for multivariate modeling, during which all parameters are concurrently varied. This approach ensures the examination of every conceivable combination of parameters with one another. The simulated input data ranges are as follows: *sy* ranging from 2 to 20 mm, *sl* ranging from 5 to 27 mm, and *sw* ranging from 1 to 6 mm.

The aggregated data from simulations and parametric sweeps are exported to MATLAB R2020b for further analysis. These data are systematically formatted to extract essential quantitative insights, including resonant frequencies, bandwidths, and peak depths. Using the obtained dataset, relationships between the output and input parameters are developed through a unified multivariate equation. In order to accomplish this, multivariate polynomial regression is employed on the data collected from the simulations. In order to evaluate the effectiveness and precision of the derived equations, the coefficient of variation of mean absolute error (CVMAE) is utilized. This measure is particularly adept at detecting overfitting due to its heightened sensitivity. The entire procedure is iteratively conducted for each output parameter, yielding a total of three distinct multivariate equations. The finalized equations with only major contributing coefficients included for brevity are presented as follows:

1 $$\begin{aligned} PD\left( {sy,~sl,~sw} \right) & = 2.463 \times 10^{{ - 3}} \left( {sy} \right)^{2} \left( {sl} \right)\left( {sw} \right)^{2} + 2.410\left( {sy} \right)\left( {sw} \right) + 0.821\left( {sy} \right)\left( {sl} \right) \\ & \quad - 0.104\left( {sy} \right)\left( {sl} \right)\left( {sw} \right) + 0.406\left( {sl} \right)^{2} \cdots \\ \end{aligned}$$

2 $$\begin{aligned} BW\left( {sy, sl, sw} \right) & = - 3.045 \times 10^{1} \left( {sy} \right)\left( {sl} \right) + 1.558\left( {sl} \right)^{2} \left( {sy} \right) - 14.68\left( {sl} \right)^{2} + 215.2\left( {sl} \right) \\ & \quad + 9.564 \times 10^{ - 1} \left( {sl} \right)^{2} \left( {sw} \right) + 3.017 \times 10^{ - 1} \left( {sl} \right)^{3} \cdots \\ \end{aligned}$$

3 $$\begin{aligned} RF\left( {sy, sl, sw} \right) & = 1.887 + 6.170 \times 10^{ - 2} \left( {sl} \right) - 1.519 \times 10^{ - 3} \left( {sl} \right)^{2} + 4.100 \times 10^{ - 2} \left( {sy} \right) \\ & \quad + 5.756 \times 10^{ - 2} \left( {sw} \right) + 2.352 \times 10^{ - 3} \left( {sy} \right)^{2} \cdots \\ \end{aligned}$$

Substituting Eqs. (1)–(3) into the fitness function which seeks to minimize itself when the output parameters converge toward the desired values. This fitness function, denoted as $F\left( {x_{1} ,x_{2} } \right)$, takes the general form displayed below:

4 $$F\left( {x_{1} ,x_{2} } \right) = \sqrt {M\left( {O_{1} - O_{1} \left( {x_{1} ,x_{2} } \right)} \right)^{2} + N\left( {O_{2} - O_{2} \left( {x_{1} ,x_{2} } \right)} \right)^{2} }$$

where *M*: Contribution factor for $$O_{1}$$, *N*: Contribution factor for $$O_{2}$$, $$O_{n} \left( {x_{1} ,x_{2} } \right)$$: Equation of first output ($$O_{n}$$) in terms of optimizing variable ($$x_{1} ,x_{2} )$$, $$O_{n}$$: Target value for n output.

Building upon this foundation, the fitness function pertaining to the output parameters can be derived as shown below:

Multivariate fitness function:

5 $$F_{M} = \sqrt {\begin{array}{*{20}c} {L\left( {BW - BW\left( {sy,sl,sw} \right)} \right)^{2} + } \\ {M\left( {PD - PD\left( {sy,sl,sw} \right)} \right)^{2} } \\ { + N\left( {RF - RF\left( {sy,sl,sw} \right)} \right)^{2} } \\ \end{array} }$$

where *L*: Contribution factor for bandwidth, *BW*: Target value for bandwidth, *M*: Contribution factor for peak depth, *PD*: Target value for peak depth, *N*: Contribution factor for resonant frequency, *RF*: Target value for resonant frequency.

**Fig. 3:**
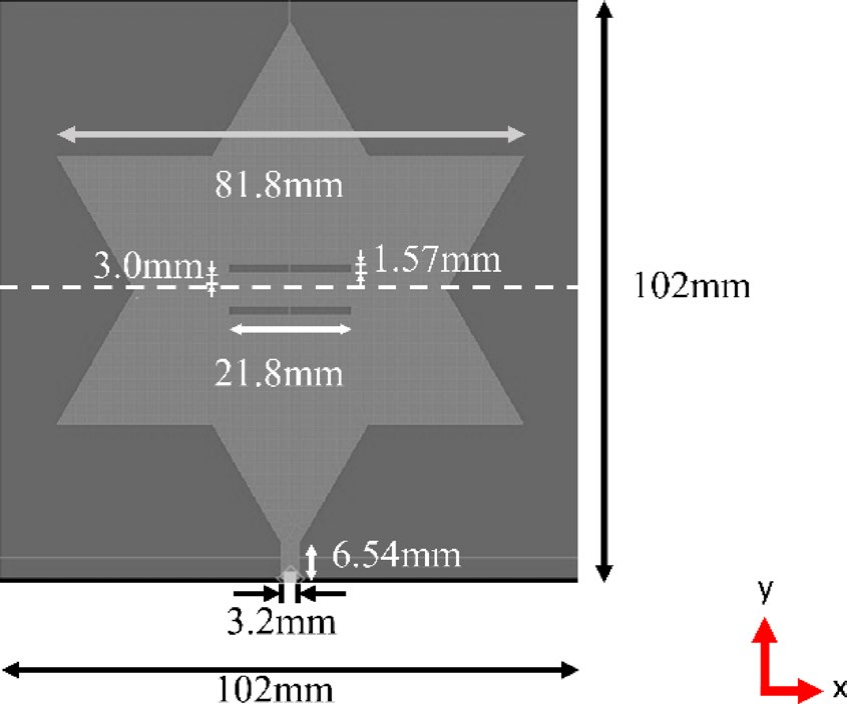
Dimension of the optimized Koch snowflake fractal patch antenna.

The resultant cost function is designed to be minimized at the predetermined target output values. Consequently, when the PSO technique is employed, the parameter values corresponding to the minima of this cost function are anticipated to represent the optimal performance points. The antenna model is optimized with the twin objectives of maximizing both bandwidth and peak depth, while simultaneously achieving a resonant frequency of 2.11 GHz. By implementing the PSO approach, the parameter values are successfully refined, leading to optimized performance. The obtained parameters are showcased in Fig. 3.

## Description of the rectifier circuit

### Input impedance of rectifier circuit

To complete the rectenna system, a rectifier circuit is designed. Consequently, a half-wave rectifier topology is chosen, owing to its simplicity and minimal component count, thereby mitigating potential losses. The SMS7630 diode is selected due to its well-documented efficiency in handling low RF input power levels^14,15^. The rectenna is designed to accommodate a broad input power range spanning from − 40 to 0 dBm. The impedance of the rectifier circuit is simulated using ADS. Notably, two distinct approaches are employed to extract the input impedance, both yielding consistent outcomes. The first approach involves the use of harmonic balance simulation—a frequency-domain analysis technique suitable for modeling non-linear circuits^16^. Given the inherent non-linearity of diodes, this technique is suitable. By using the current probe at the junction between the input port and the rectifier circuit, the input current (*I*_*in*_) is captured alongside the input voltage (*V*_*in*_) across the circuit. Utilizing Ohm’s law as shown in Eq. (6), the input impedance of the rectifier circuit is calculated.

6 $$Z = \frac{{V_{in} }}{{I_{in} }}$$

**Fig. 4:**
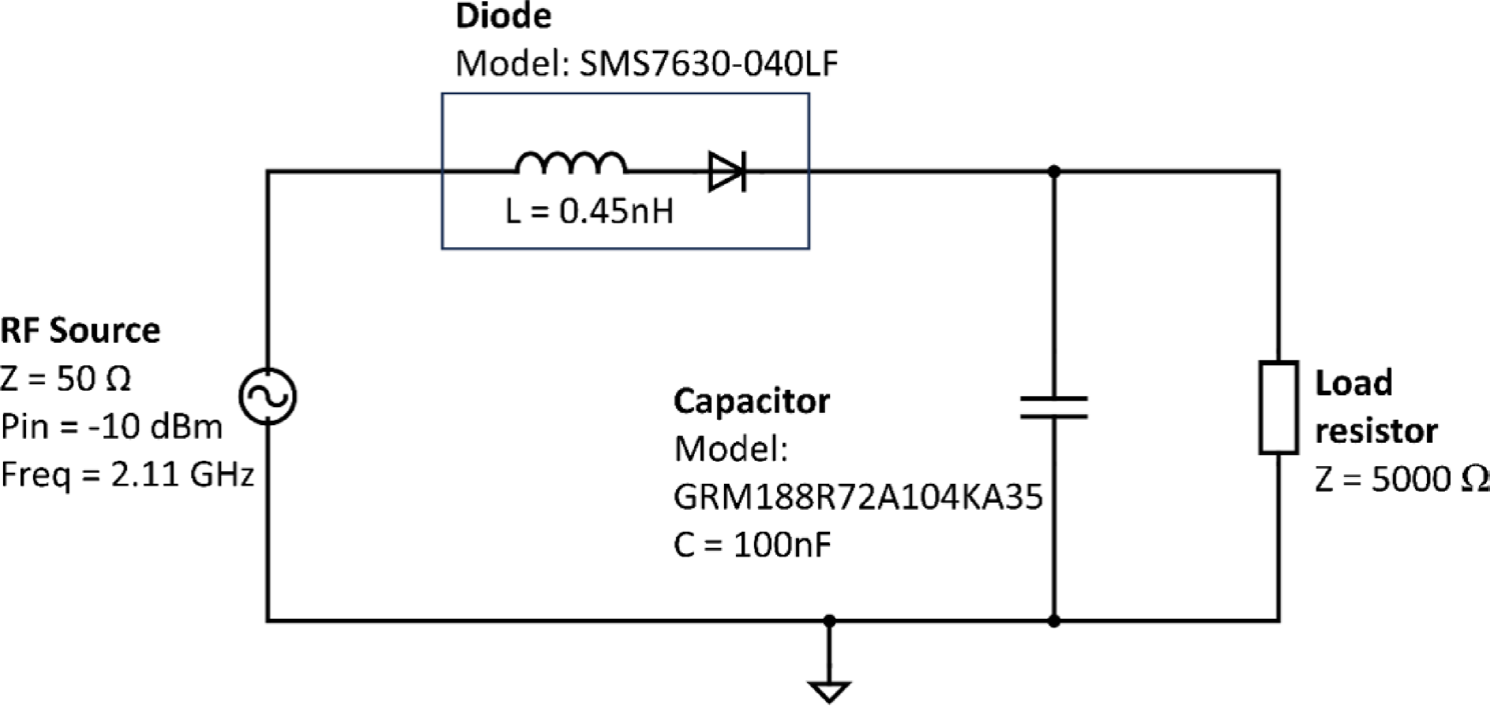
The input impedance of the rectifier circuit setup.

Alternatively, the second method utilizes the large signal S-parameter (LSSP) function within ADS. This methodology suits the non-linear nature of the rectifier circuit, which variably responds to input power levels. By employing both LSSP and Zin functions to the circuit as depicted in Fig. 4, the input impedance can be derived. The Zin function, reliant on the reflection coefficient and reference impedance, measures the input impedance when looking into the measurement ports. A simple single-stage rectifier circuit incorporating the SMS7630 diode model is constructed, while the input impedance simulation setup is outlined in Fig. 4. The characteristics of the diode are parameterized using SPICE model parameters extracted from Skyworks’ datasheet^17^. Additionally, an inductor is introduced to account for the series inductance inherent in the diode package, as stated in the datasheet.

**Fig. 5:**
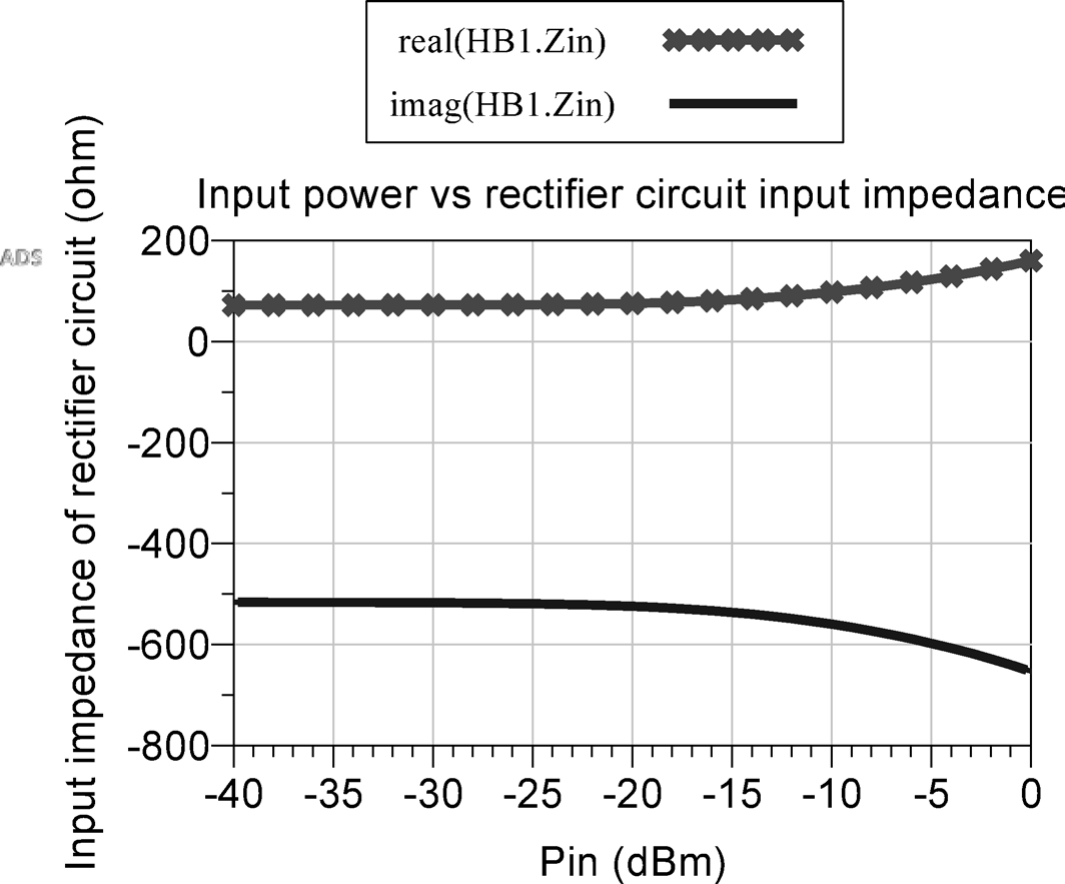
Rectifier circuit input impedance as a function of input power.

Hence, the input impedance of the selected rectifier circuit, evaluated as a function of input power at the resonant frequency of the antenna (2.11 GHz), is simulated utilizing the LSSP analysis function and the Zin function within the ADS software. The real and imaginary components of the input impedances of the rectifier are extracted and graphed over the power range of − 40 to 0 dBm, as visually depicted in Fig. 5. Observing the figure, it becomes evident that the real component of the input impedance of the rectifier circuit exhibits a steady increase, ranging from 72.105 to 159.804 Ω, while the imaginary component undergoes a corresponding decrease, spanning from − 516.387 to − 652.864 Ω, as the input power increases.

### Matching network

**Fig. 6:**
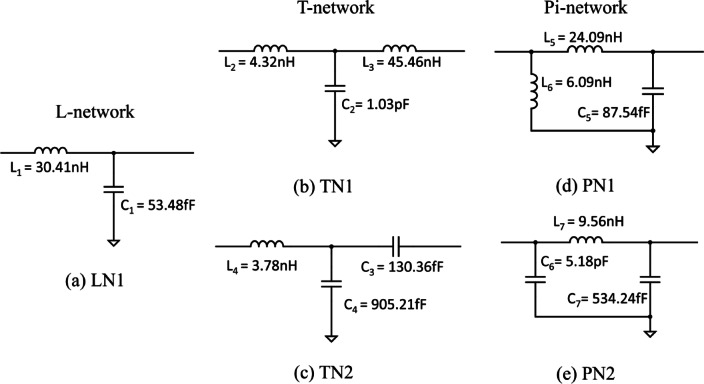
(**a**) L-network configuration (LN1). (**b**) T-network with a series capacitor connected to the selected L-network (TN1). (**c**) T-network with a series inductor connected to the selected L-network (TN2). (**d**) Pi-network with a shunt inductor connected to the selected L-network (PN1). (**e**) Pi-network with a shunt capacitor connected to the selected L-network (PN2).

The impedance-matching network partly determines the RF-DC efficiency of the rectenna. In a matched circuit, the maximum amount of power can be transferred between the source and the load. Therefore, the antenna impedance, which is determined to be 50 Ω, is matched with the input impedance of the rectifier circuit obtained through the ADS simulation. Common matching network configurations comprised of the L, Pi, and T networks. In the context of the L-network, eight potential configurations exist^18^. In order to facilitate a low-pass configuration for direct current (DC) passage into the load, the inductor needs to be in series connection. Furthermore, if the load impedance is greater than the source impedance, while being positioned to the right, the parallel component of the L-matching network must be situated on the right side. Addressing these prerequisites narrows down the options to a single viable configuration—a series inductor coupled with a shunt capacitor, depicted in Fig. 6a.

Subsequent advancements are undertaken to derive T and Pi networks, building upon the selected L-network as the base configuration. For the T-network, the introduction of a series capacitor or inductor on the right side of the L-network is exemplified in Fig. 6b,c. Conversely, a shunt inductor or capacitor is accommodated on the left side of the L-network, resulting in a Pi-network configuration, showcased in Fig. 6d,e.

These five configurations are subject to optimization and simulation within ADS, aimed at identifying the optimal matching network. The initial determination of component values for the matching circuits is facilitated using the ADS Smith chart utility. The Smith chart, a graphical tool, provides insight into the impedance variations of transmission lines and antenna systems over frequency. It is utilized to design an impedance-matching network by moving the load impedance to meet the source impedance on the chart, achieved through the addition of lumped components such as capacitors and inductors. Upon successful impedance matching, the resultant matching network is integrated into the rectifier circuit. Subsequently, the circuit undergoes simulation utilizing the harmonic balance technique within ADS, enabling the determination of both the output power of the circuit and the RF-DC efficiency.

The results of the five proposed matching networks show that the TN1 matching network presented an inherent challenge. The critical issue lay in the unavailability of a combination of inductor and capacitor values capable of achieving the desired impedance match over the intended frequency range. Consequently, this led to the exclusion of the TN1 network from further consideration. The remaining four matching networks (LN1, PN1, PN2, and TN2) underwent an optimization process aimed at realizing peak efficiency.

### Optimization

**Fig. 7:**
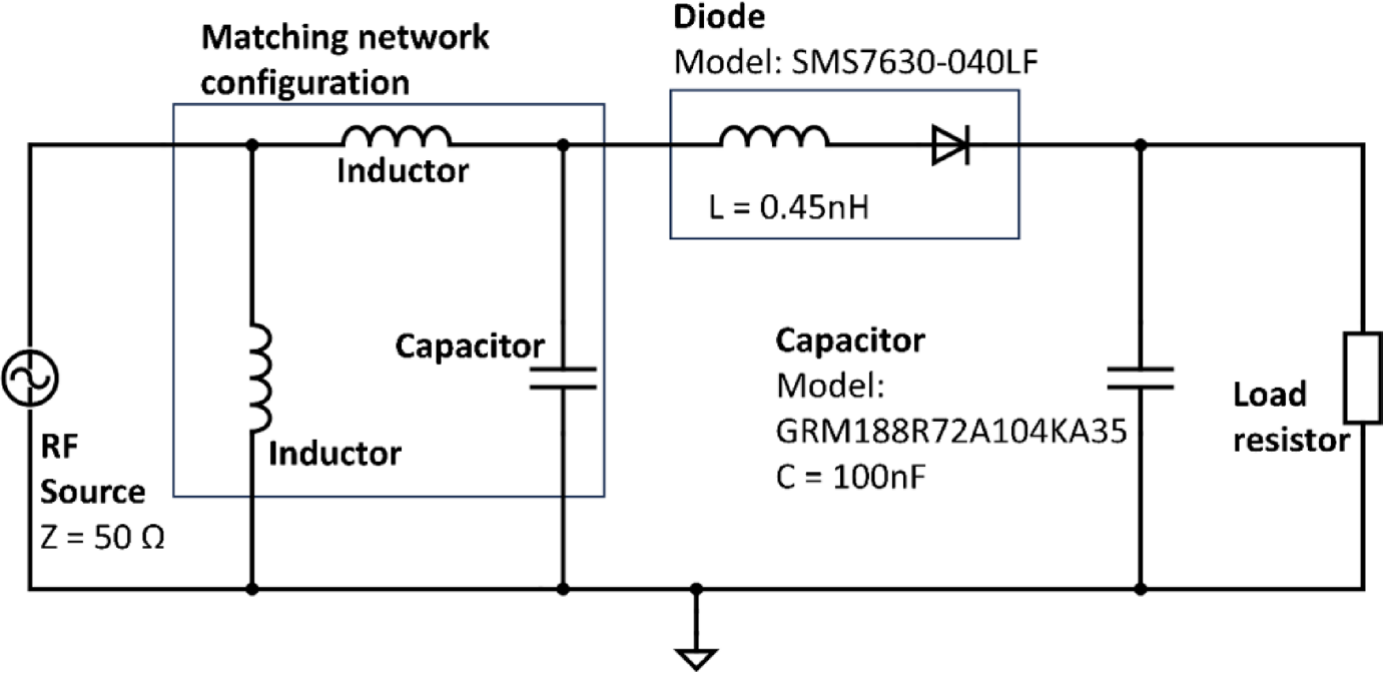
Circuit setup for optimization.

The subsequent optimization is executed utilizing the optimization function within ADS. This optimization process is executed to refine the component values. The process is initiated by configuring the optimization goals to the circuit as illustrated in Fig. 7. The optimization objectives encompassed two pivotal aspects: enhancing the peak depth of the return loss and the RF-DC efficiency of the circuit. Harmonic balance simulations are conducted through a parameter sweep across varying input power levels. This approach enabled the generation of plots portraying output power and RF-DC efficiency as functions of input power. The final optimization shows that PN1 as the best candidate. The matching network results can be found in Result and discussion.

Upon concluding the optimal matching network configuration, the specific values for inductors and capacitors are precisely identified. These optimized values are required for the selection of actual component models and corresponding values. Murata Manufacturing, a Japanese manufacturer of electronics components, is one of the vendors that is considered for the selection of the components. Subsequently, the S-parameter files corresponding to these components are sourced from the vendor. This approach facilitated the inclusion of vendor components into ADS simulations, ensuring that the simulated performance closely mirrors the anticipated real-world circuit behavior. The components utilized in conjunction with their respective models are tabulated in Table 1.

**Table 1:** Component models and values selected.

Component	Manufacturer	Model	Value
Inductor 1 (L1)	muRata electronics	LQW15AN4N6B00D	4.6 nH
Inductor 2 (L2)	muRata electronics	LQW04AN22NH00D	22 nH
Capacitor 1 (C1)	KEMET	CBR04C108B1GC	0.1 pF
Capacitor 2 (C3)	muRata electronics	935,151,424,610	100 nF
Diode (D1)	Skyworks Solutions Inc	SMS7630-040LF	–
Load resistor (R1)	Walsin Technology	WR08X3301FTL	3.3 kΩ

### Fabrication

**Fig. 8:**
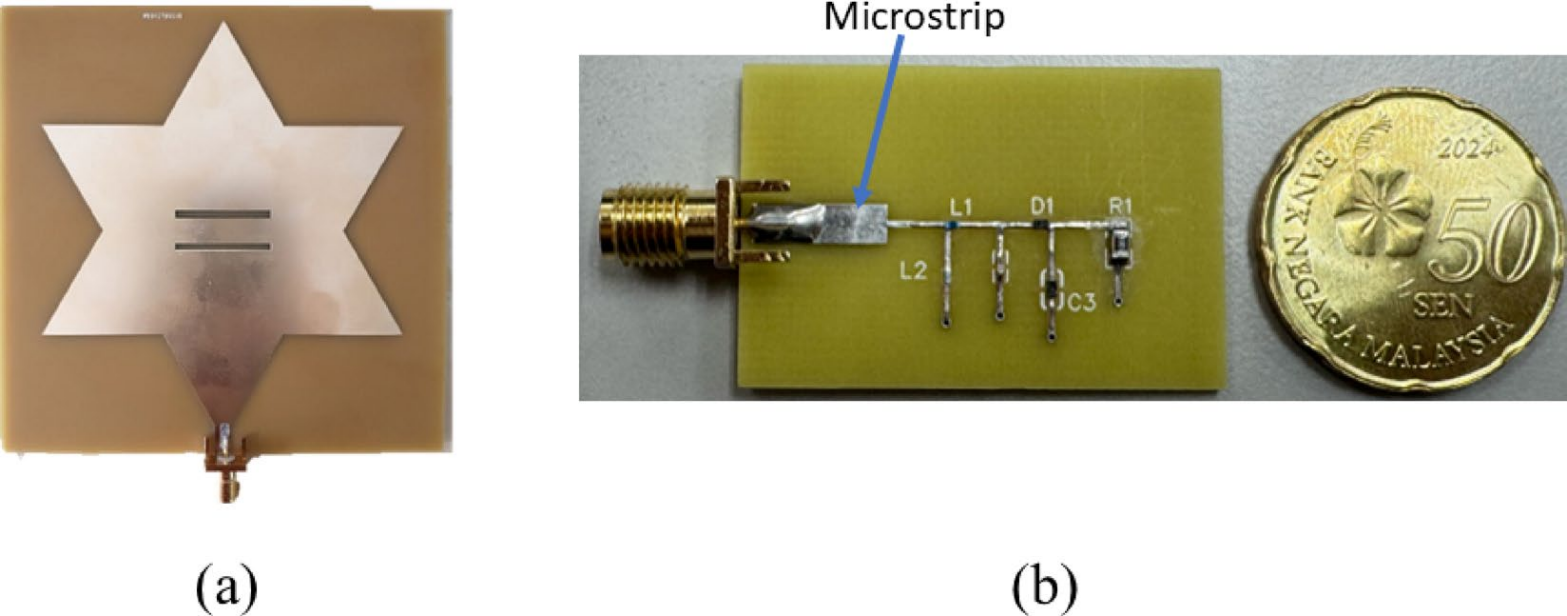
(**a**) Fabricated Koch snowflake fractal patch antenna. (**b**) Fabricated rectifier circuit with Malaysia 50 Sen coin beside to show its relative size. L1, L2 are inductor 1 and inductor 2 respectively, C1 and C3 are capacitor 1 and capacitor 2 respectively, D1 is the diode and R1 is the load resistor as specified in Table 1.

A few PCB specifications must be considered during the ordering process such as the size, layers, material used, thickness of the board, surface finishing, copper thickness, and etc. The dimension of the antenna is 102 mm × 102 mm whereas the rectifier circuit is 24 mm × 36 mm. For the base material of the PCB, the cost-efficient option of FR-4, a composite incorporating flame-retardant epoxy resin and glass fabric, is used. The substrate thickness for both antenna and rectifier circuit is 1.6 mm while the copper thickness is 35 microns. A 50-Ω microstrip line is incorporated for impedance matching between the SMA connector and the circuit. The width of the microstrip line is calculated using a microstrip impedance calculator, obtaining a width of 2.94 mm and a length of 10 mm. The resulting fabricated antenna is depicted in Fig. 8a, while Fig. 8b showcases the fabricated rectifier circuit.

## Results and discussion

### Matching network

**Fig. 9:**
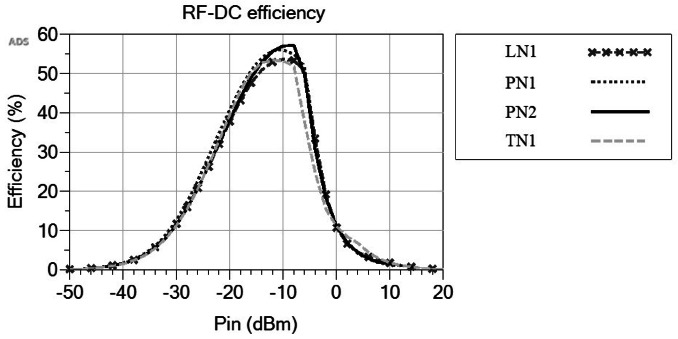
RF-DC efficiency for the four matching networks.

Figure 9 illustrates the RF-DC efficiency of the rectifier circuit utilizing the four different matching networks. Each network exhibits an efficiency peak at approximately − 10 dBm, aligning with the input power level for which the circuits are optimized. Furthermore, the graph demonstrates that the rectifier circuit consistently attains an RF-DC efficiency exceeding 10% across the input power spectrum ranging from − 30 to 0 dBm. Upon close examination, it becomes evident that the Pi networks outperform the L and T networks, particularly within the − 20 to 0 dBm input power range. The PN1 network demonstrates greater efficiency from − 40 dBm to − 12 dBm, whereas the PN2 network exhibits better performance within the − 12 to − 7 dBm range. In contrast, both networks perform comparably at higher input power levels. Therefore, the PN1 network emerges as the preferred choice for the matching network of the rectifier circuit. It offers higher efficiency over a broader range of input power levels compared to the PN2 network. The PN1 network attains a peak efficiency of approximately 55% at an input power of − 11 dBm.

### Antenna

**Fig. 10:**
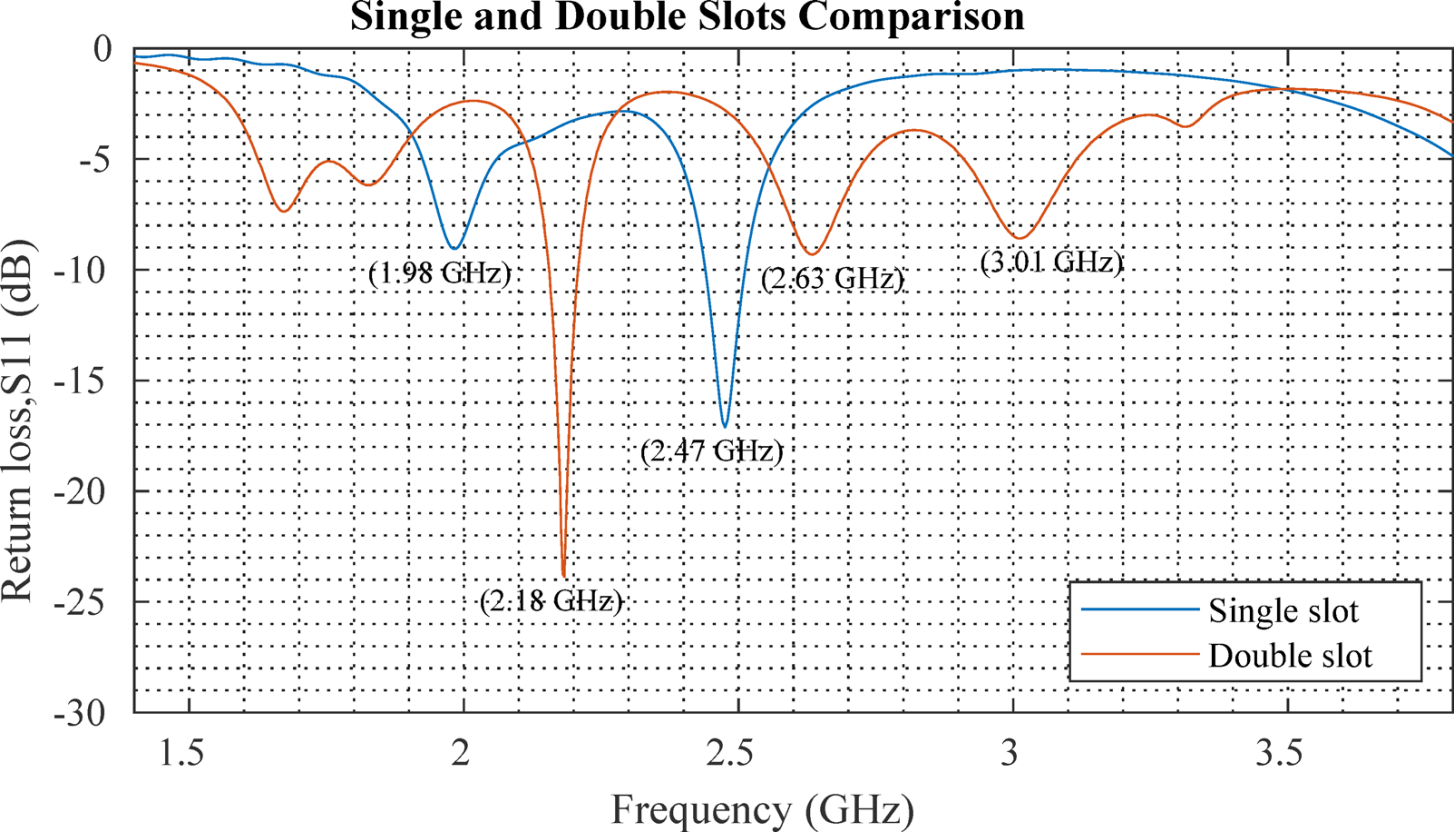
Comparison of (simulated) return loss for single and double rectangular slot Koch fractal antennas.

**Fig. 11:**
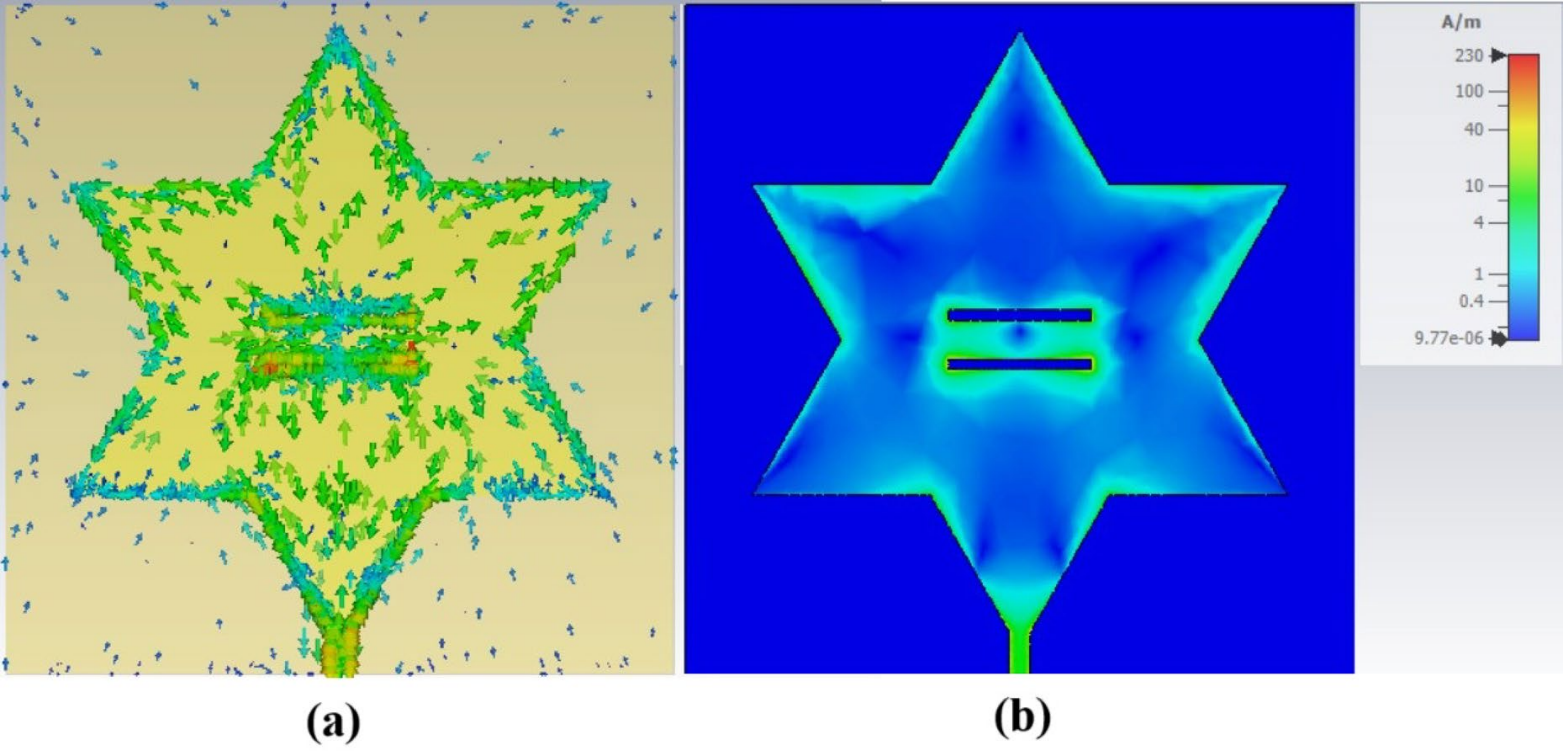
Simulated surface current plots for optimized double-slot antenna illustrated via arrows (**a**) and contours (**b**).

The comparison between single-slot and double-slot Koch fractal antennas, as illustrated in Fig. 10, demonstrates that the double-slot antenna outperforms the single-slot design, particularly in terms of additional resonance modes and deeper return loss peaks. The single-slot antenna primarily resonates at 1.98 GHz and 2.47 GHz, exhibiting a relatively shallower return loss. In contrast, the double-slot antenna introduces a strong resonance at 2.18 GHz, along with additional resonances at 2.63 GHz and 3.01 GHz, highlighting its multiband characteristics and enhanced impedance matching. These improvements can be attributed to the increased current distribution complexity and enhanced capacitive and inductive coupling that is introduced from the addition of the optimized double slot, as seen in Fig. 11. As discussed by Parikh and Singh^11^, introducing slots in a microstrip patch antenna perturbs the surface current flow, leading to frequency shifts and the emergence of multiple resonance modes. The observed results align with this principle, demonstrating that the double-slot configuration provides greater frequency selectivity and impedance matching, making it more suitable for applications requiring efficient multiband operation. The deeper return loss observed in the double-slot design indicates that the antenna can efficiently transfer power, reducing reflection losses and enhancing overall radiation efficiency. The findings confirm that optimizing slot configurations can significantly improve antenna performance without requiring complex fractal shape with higher fractal iterations, making it a practical approach for enhancing rectenna designs.

**Fig. 12:**
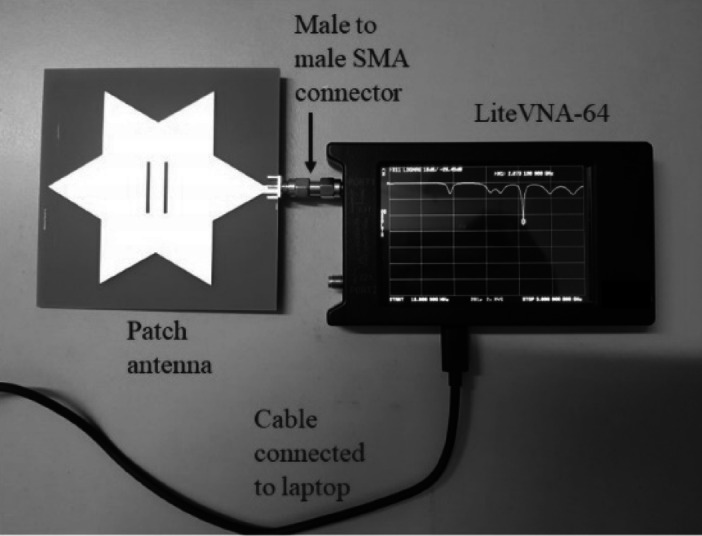
Measurement setup to measure the return loss of the antenna.

**Fig. 13:**
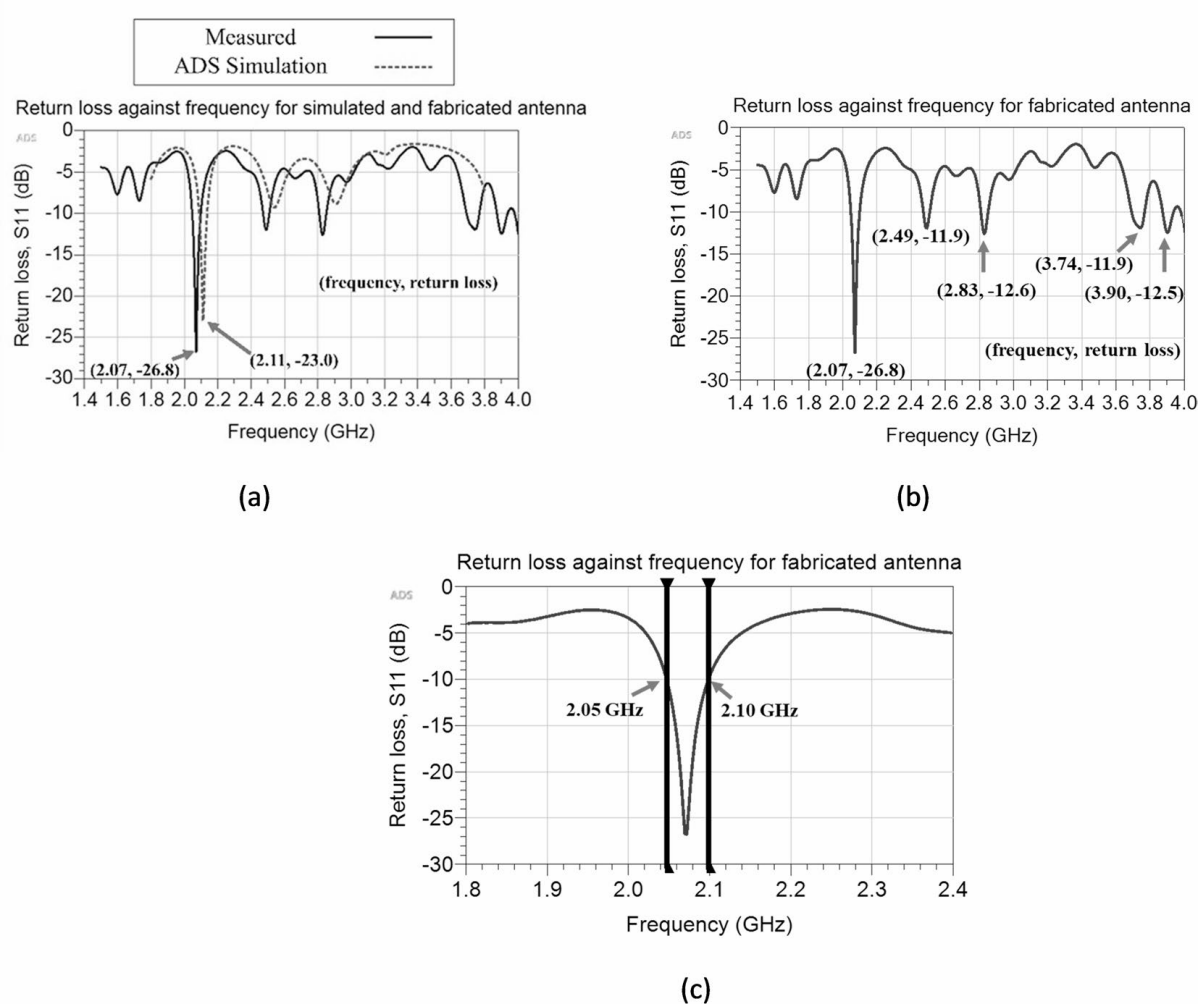
(**a**) Simulated and measured return loss of the antenna. (**b**) Return loss graph of the fabricated antenna showing the multiband characteristics. (**c**) Zoom-in view of the return loss graph of the fabricated antenna within the frequency range of 1.8 GHz to 2.4 GHz.

The performance of the fabricated antenna is evaluated based on its return loss. The return loss of the antenna is measured using a vector network analyzer (VNA), specifically the LiteVNA-64, a portable and cost-effective VNA unit spanning a frequency range from 50 kHz to 6.3 GHz. The experimental setup is shown in Fig. 12, where the VNA interfaces with the antenna via a male-to-male SMA connector. A return loss graph, as illustrated in Fig. 13a, showcases the return loss trends of the simulated antenna using ADS software, as well as the return loss of the fabricated antenna. Notably, the simulated agrees well with the measured results. Within the frequency range spanning from 2 to 3 GHz, three distinct peak depths are discernible. The resonant frequency of the fabricated antenna with the deepest peak depth is at 2.07 GHz, with a return loss value of − 26.8 dB. In contrast, the ADS simulation result shows the resonant frequency at 2.11 GHz with a return loss of − 23.0 dB. In light of these findings, it is shown that the return loss of the fabricated and simulated antenna is in reasonable agreement. The slight deviation in the resonant frequency of the fabricated antenna may be attributed to factors such as the insertion loss of the SMA connector, an aspect not explicitly considered during the simulation.

**Fig. 14:**
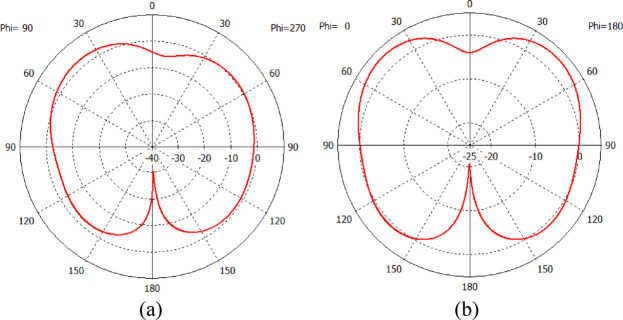
(**a**) Simulated 2D radiation pattern in E-plane. (**b**) Simulated 2D radiation pattern in H-plane.

**Fig. 15:**
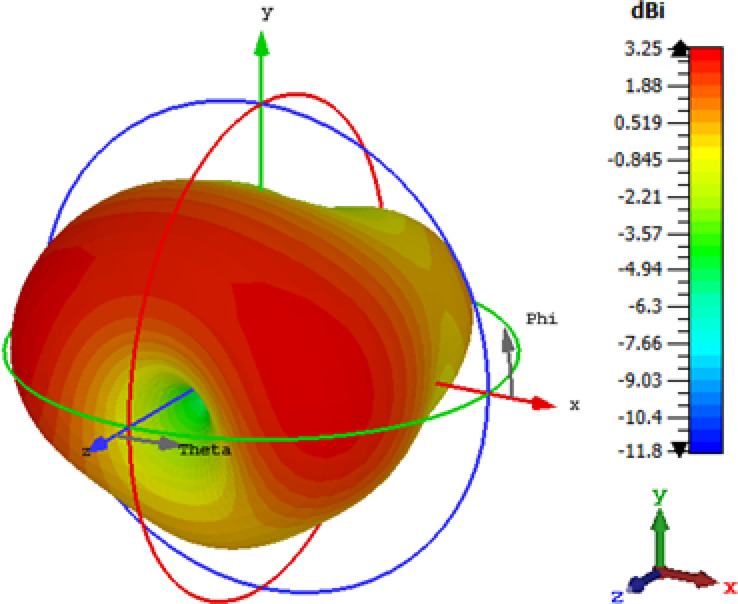
Simulated 3D radiation pattern using Computer Simulation Technology (CST) Studio Suite 2019 software (https://www.3ds.com/products/simulia/cst-studio-suite).

As demonstrated in Fig. 13b, the return loss graph of the fabricated antenna reveals intriguing multiband characteristics. Within the frequency spectrum ranging from 1.7 to 4 GHz, there are a total of five distinct peak depths. These peaks occur at specific frequencies: 2.07 GHz, exhibiting a magnitude of − 26.8 dB; 2.49 GHz, with a magnitude of − 11.9 dB; 2.83 GHz, with a magnitude of − 12.6 dB; 3.74 GHz, exhibiting a magnitude of − 11.9 dB; and 3.90 GHz, with a magnitude of − 12.5 dB. For a closer examination of the first peak depth, Fig. 13c presents a zoom-in view of the return loss graph within the frequency range of 1.8 GHz to 2.4 GHz. Notably, the bandwidth of the antenna spans approximately 52 MHz, from 2.05 to 2.10 GHz, where the return loss consistently maintains values below − 10 dB. In addition, Fig. 14a,b illustrate the simulated radiation pattern of the antenna in 2D, showing the E-plane and H-plane respectively at 2.07 GHz. The 3D radiation pattern can be found in Fig. 15, demonstrating the overall well-receiving capability of the antenna for harvesting RF energy.

A comparison among the proposed antenna and relevant antenna designs is provided in Table 2. The selected references for comparison have been carefully chosen to closely resemble the design of the proposed antenna. The proposed antenna exhibits superior performance in terms of multiband functionality and return loss compared to the work by Dandgavhal et al.^19^ and Kharat et al.^20^, as presented in Table 2. In comparison to the work presented by Annou et al.^21^, the proposed antenna excels in multiband functionality and maintains a competitive position in terms of return loss, albeit with a trade-off in bandwidth. Remarkably, despite being only at the 1st iteration, the proposed antenna yields results that are on par or even superior to those of related works presented in references^19–21^. This underscores the simplicity and ease of fabrication of the proposed antenna. It is worth noting that while the related works are based on simulations, the proposed antenna performance is validated through measurement.

**Table 2:** Comparisons between the proposed antenna and some related works.

Ref	Antenna type	Iterations	Resonant freq. (GHz)	Return loss (dB)	Bandwidth (MHz)
^14^	Koch snowflake fractal patch with complimentary split ring resonator	2nd	2.66	− 25.9	–
			5.62	− 9.5	–
^22^	Koch snowflake fractal patch	2nd	1.24	− 11.9	7.9
			1.42	− 19.2	10.4
			2.91	− 11.9	41.2
^13^	Suspended Koch snowflake fractal patch	3rd	2.46	− 15.2	246.0
			3.53	− 11.3	120.0
			3.88	− 20.9	257.0
This work	Koch snowflake fractal patch with double rectangular slots	1st	2.07	− 26.8	52.0
			2.49	− 11.9	36.0
			2.83	− 12.6	39.0
			3.74	− 11.9	76.0
			3.90	− 12.5	61.0

### Rectifier circuit

In evaluating the performance of the fabricated rectifier circuit, the LiteVNA-64 vector network analyzer is also used to measure the return loss of the circuit. The currently reported circuit is an improvement of the initial design. The initial circuit exhibited a drastic frequency shift due to several factors. One key reason was the absence of a microstrip line between the SMA connector and the circuit, leading to impedance mismatch. Moreover, parasitic capacitance exerts a substantial influence on circuit performance and is closely tied to the self-resonant frequency (SRF) of the capacitor^23^. The SFR is the frequency at which the capacitor stops exhibiting typical capacitive behavior and starts to exhibit inductive characteristics. Therefore, the higher the SRF of the capacitor compared to the operating frequency of the circuit, the lesser the parasitic effects it introduces. In the previous design, Capacitor 2 had an SRF of 23 MHz, which is substantially lower than the operating frequency of 2.07 GHz, contributing to performance degradation. To address these issues in the modified circuit, a 50-Ω microstrip line (width: 2.94 mm, length: 10 mm) is introduced for impedance matching. Additionally, Capacitor 2 is replaced with the model: 935151424610, ultra-broadband silicon capacitor, capable of operating up to 60 GHz, as listed in Table 1.

**Fig. 16:**
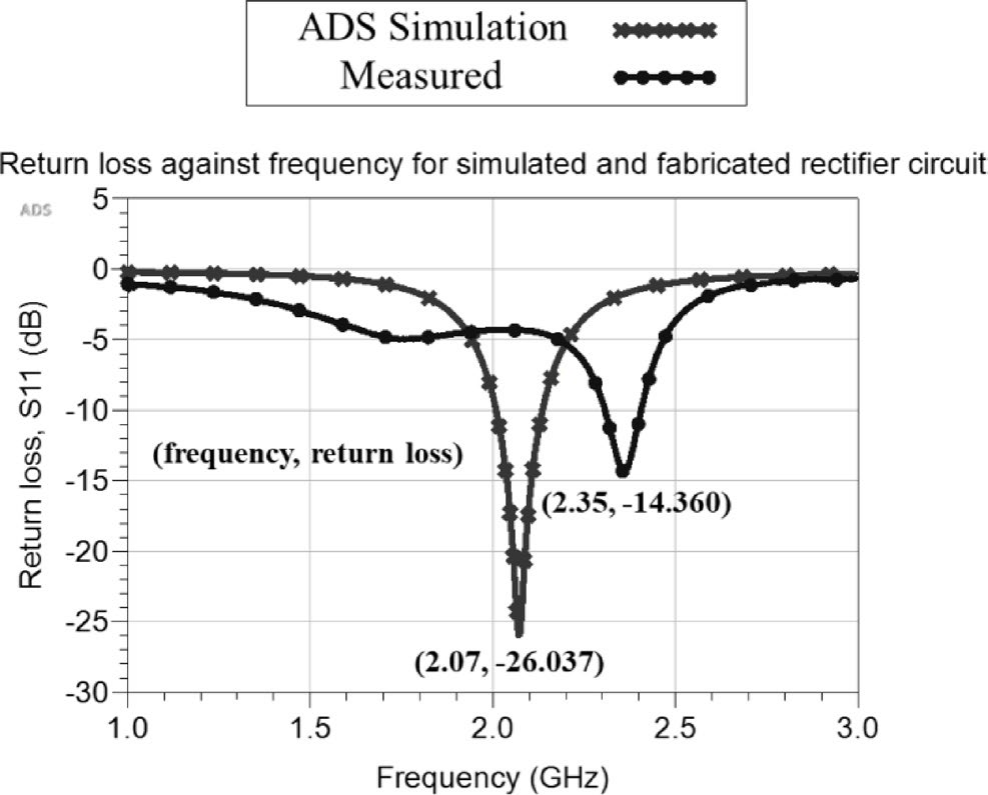
Simulated and measured return loss of the rectifier circuit.

A comparison between the simulated and measured return loss of the rectifier circuit is presented in Fig. 16. The fabricated rectifier circuit exhibits an optimal frequency of 2.35 GHz, closely matching the simulated 2.07 GHz, thereby significantly reducing the frequency shift observed in the previous design. However, the return loss increased from -26.0 dB in the simulation to − 14.36 dB in the measurement, indicating higher reflected power. While the resonant frequency alignment has notably improved, a minor frequency shift is still observed. Future work can incorporate the capacitive loading method by adding capacitors to the rectifier circuit to shift the frequency downward^24^. Specifically, incorporating a varactor with variable capacitance could offer a dynamic tuning solution, as demonstrated in the previous study^25^. This approach enables adjustable impedance matching between the antenna and rectifier circuit, addressing potential mismatches that may arise due to fabrication tolerances or varying load conditions. Additionally, the PCB layout can be further optimized by minimizing unnecessary trace lengths and eliminating sharp bends to improve impedance matching and overall circuit performance.

**Fig. 17:**
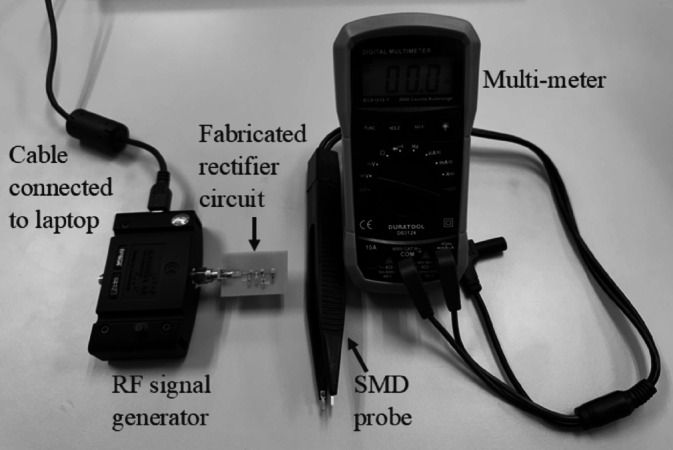
Measurement setup to measure the RF-DC efficiency of the rectifier circuit.

Another performance metric, RF-DC efficiency, is also assessed in this paper to evaluate the performance of the rectifier circuit. The Aaronia BPSG6 RF signal generator, as depicted in Fig. 17, with a frequency range spanning from 23.5 MHz to 6 GHz and an input power scope from − 45 to 18 dBm is utilized in this experiment. Figure 17 visually represents the setup employed. This RF signal generator functioned as a controlled source of RF power, facilitating precise testing and measurement of the performance of the rectifier circuit. The RF signal generator interfaced with a laptop containing the device program. This program enabled manual configuration of the desired frequency and input power levels to be emitted by the generator. Accordingly, the RF signal generator is configured at the frequency at which the rectifier circuit demonstrated optimal performance. Through systematic variation of input power levels from -40 dBm to 18 dBm, with a 1 dBm increment, at this fixed frequency, the RF-DC efficiency of the rectifier circuit could be assessed under varying operational conditions. At each adjusted input power level, the multi-meter captured the output voltage of the rectifier circuit. Subsequently, the RF-DC efficiency of the rectifier circuit is calculated using Eq. (7):

7 $$RF - DC_{eff} = \frac{{V_{out}^{2} }}{{P_{in} *R_{load} }}*100 \%$$

where $V_{out}$: Output voltage at the load resistor, $R_{load}$: Resistance of load resistor, $P_{in}$: Input power.

**Fig. 18:**
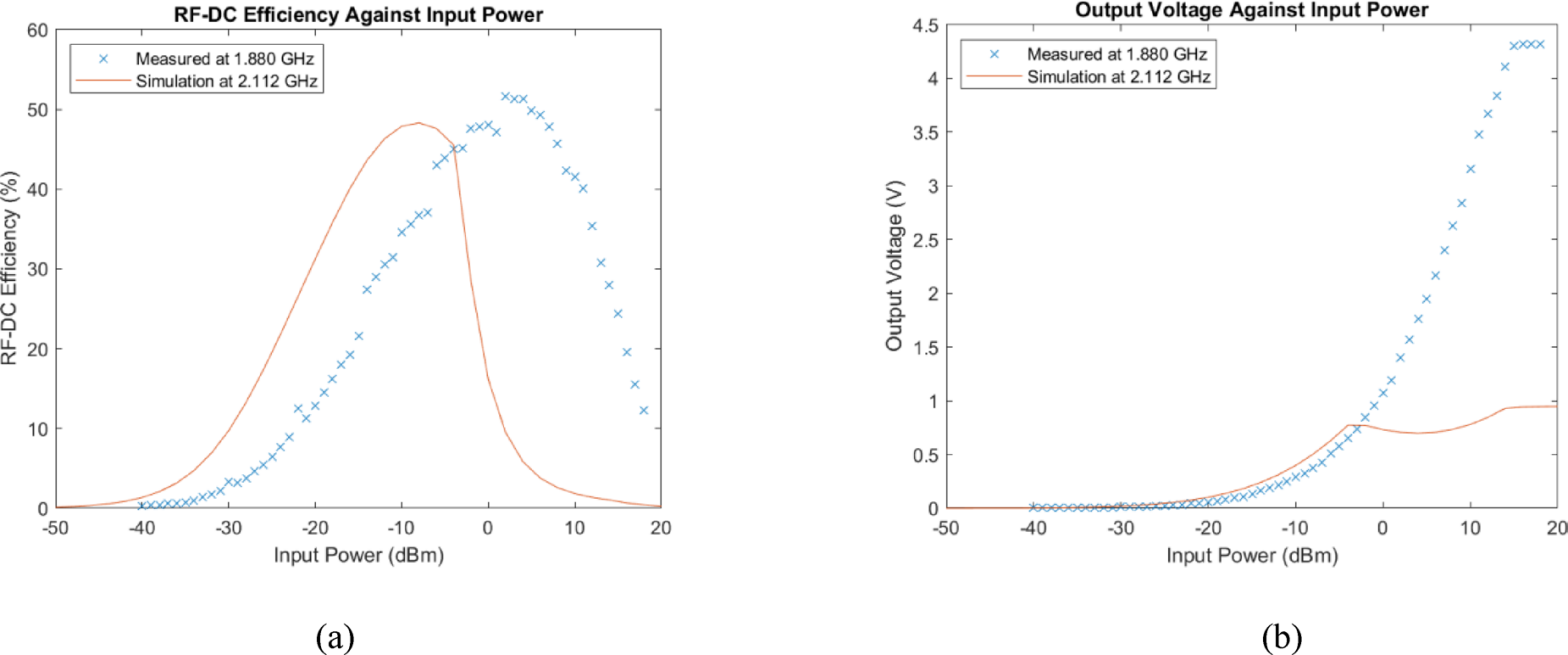
(**a**) Measured and simulated RF-DC efficiency of the rectifier circuit against input power. (**b**) Measured and simulated output voltage of the rectifier circuit against input power.

Initially, the RF-DC efficiency of the rectifier circuit is measured at a fixed input power of 5 dBm over a frequency range spanning 1.1 GHz to 3 GHz. The objective is to determine the frequency range where the rectifier circuit attains its optimal performance. This refined analysis pinpoints the peak efficiency at approximately 1.88 GHz. Figure 18a delivers a comparative view of the measured and simulated RF-DC efficiencies of the rectifier circuit as they vary with input power. It is important to emphasize that the measured efficiency is recorded at 1.88 GHz, while the simulated efficiency is performed at 2.11 GHz, a frequency relatively close to the measured peak. In the RF-DC efficiency measurement, the circuit operates at − 40 dBm, which marks the minimum activation power required for the rectifier circuit to commence operation. As the input power increases, the efficiency exhibits gradual increments until it reaches a peak efficiency of 51.7% at 2 dBm. Subsequently, beyond 5 dBm, a significant decline in efficiency is observed, attributed to the output voltage exceeding the breakdown voltage threshold of the SMS7630 diode. This higher input power regime results in a degradation of the performance of the rectifier circuit. Figure 18b provides a corresponding representation of the output voltage trends, showcasing gradual increments from − 40 to around 15 dBm, after which it saturates at 4.3 V.

The observed difference between the optimal return loss, S11 at 2.35 GHz, and the peak RF-DC efficiency at 1.88 GHz in the rectifier circuit arises from distinct factors governing impedance matching and power conversion. Impedance matching networks, such as the L-section used in Cockcroft-Walton multipliers, are optimized to minimize reflections at specific frequencies by transforming the antenna impedance to the input impedance of the rectifier circuit^26^. However, even if S11 is minimized at 2.35 GHz, the conversion efficiency of the diode depends on nonlinear diode behavior, harmonic interactions, and parasitic elements, which can shift the optimal operating frequency. For instance, parasitic capacitances in Schottky diodes and PCB vias introduce frequency-dependent losses, degrading efficiency at higher frequencies despite good impedance matching^23,27^.

Moreover, the simulation assumes ideal transmission lines, whereas in practice, transmission line characteristics significantly impact high-frequency circuit performance. Parameters such as width, length, spacing, and edge effects introduce variations that contribute to losses^27^. To address these challenges, future work should prioritize simulating a complete rectifier circuit model that incorporates parasitic elements. This can be achieved by modeling the capacitors and inductors in the circuit as series and shunt LC tanks, while the diode should include parallel parasitic capacitance and junction capacitance as demonstrated by Alex-Amor et al.^26^. Additionally, optimizing transmission line parameters^27–29^ through EM model co-simulation using tools such as ADS Momentum will help minimize impedance mismatches and reduce transmission losses, which is critical for improving RF-DC conversion efficiency. Fabrication-related losses must also be considered, as factors such as SMA connector mismatches, solder joint imperfections, SMD chip tolerances, and microstrip discontinuities can contribute to performance deviations^28^. Future work should investigate methods to mitigate these losses and discrepancies, ensuring a more accurate and high-performance rectifier design. By addressing these factors, future work can help minimize deviations and misalignment between optimal return loss and peak RF-DC efficiency, ultimately improving the accuracy and efficiency of the rectifier circuit.

Table 3 summarizes the performance of the fabricated rectifier circuit of this work with those from other literatures, specifically in terms of RF-DC efficiency. The fabricated design can operate at a wide range of input power from − 16 to 16 dBm with an efficiency surpassing 20%. The lower limit of the input power for this design, where the RF-DC efficiency reaches 20%, is at − 16 dBm. Comparing the results with the referenced works presented in Table 3, it is evident that the fabricated design is comparable to the lower limit of Bito et al.^30^ and outperforms the other two reference works of Alex-Amor et al.^23^ and Lee et al.^31^.

**Table 3:** Comparisons between the simulated rectifier circuit and some related works.

Ref	Operation freq (GHz)	Rectifier circuit configuration	RF-DC efficiency (%)
^27^	2.45	Voltage doubler	> 20% (− 18 dBm to beyond 0dBm) Maximum efficiency—48% at 0 dBm
^21^	0.87	Half-wave Cockcroft Walton	> 20% (− 5 dBm to beyond 15 dBm) Maximum efficiency—39% at 13 dBm
^28^	2.45	Half-wave	> 20% (− 2.5 to 15 dBm) Maximum efficiency—79.96% at 10.5 dBm
This work	1.88	Half-wave	> 20% (− 16–16 dBm) Maximum efficiency—51.7.% at 2 dBm

In relation to the practical implications and potential challenges in implementing the proposed rectenna system in real-world IoT applications, the main challenges are the low power density of ambient RF energy and the variation in frequencies across different locations. This is because different places have different power sources, such as TV and radio broadcasts, cellular base stations, and Wi-Fi routers, all operating at different frequencies. However, an IoT device that utilizes RF energy harvesting as its power source should be able to function across different frequencies and locations. Therefore, a rectenna that is wideband or multiband is needed to overcome these challenges. A wideband or multiband antenna increases the chances of harvesting low power density sources and enables the device to operate in various locations. The proposed antenna solution exhibits multiband characteristics, making it suitable for integration into IoT devices. Our research initiated the incorporation of slots into the fractal antenna, identifying the optimal slot positions through a multivariate parametric modelling-based approach with particle swarm optimization to enhance the bandwidth and return loss of the antenna. However, challenges remain in efficiently harvesting low ambient power density, as efficiency must be further enhanced to ensure energy conversion without waste, given that the input power is already very small. One potential solution is to design an array of rectennas to increase efficiency, though this comes at the expense of size. This brings us to another potential challenge, the size of the rectenna, which should be small enough to fit within an IoT device without occupying excessive space. The proposed antenna can maintain its compact size while improving performance by adding slots. By addressing the challenges mentioned, the rectenna system will be a promising solution for enhancing the feasibility and efficiency of RF energy harvesting in real-world IoT applications.

In this work, a Koch snowflake fractal patch antenna was fabricated, resonating at 2.07 GHz with a return loss of − 26.8 dB. The addition of double rectangular slots to the first iteration fractal pattern enhanced antenna performance, producing deeper return loss and additional resonances, which improve the multiband functionality. Compared to the single-slot design, the double-slot configuration introduced coupled resonances, resulting in improved bandwidth. Leveraging these advantages, the optimized double-slot antenna was integrated into a rectenna system. A rectifier circuit with a Pi-matching network was designed to handle a wide input power range, achieving optimal performance at 1.88 GHz with an RF-DC efficiency exceeding 20% from − 16 to 16 dBm.

## Conclusion

This work involves the development of a rectenna system, comprising a Koch snowflake fractal patch antenna with double rectangular slots and a rectifier circuit with a Pi matching network. A comparative analysis between single and double slots demonstrates that the double-slot configuration outperforms the single-slot design. The antenna exhibits strong overall performance, validated through experiments that closely match simulations, demonstrating the feasibility of incorporating rectangular slots onto the Koch snowflake fractal patch. Future work should prioritize further antenna optimization in terms of size to fit small IoT devices. Additionally, an initial discrepancy between the fabricated and simulated rectifier circuit resonant frequencies was observed but mitigated by introducing a microstrip line for impedance matching and a capacitor with higher self-resonant frequency to minimize the effect of parasitic capacitance. This adjustment reduced signal reflections and losses, achieving an RF-DC efficiency exceeding 20% over an input power range of -16 dBm to 16 dBm at 1.88 GHz. However, this frequency is still slightly shifted from the target of 2.11 GHz. To address this, future efforts should incorporate comprehensive EM model co-simulation to enhance accuracy and achieve a closer match between the antenna and rectifier circuit resonant frequencies. Lastly, the entire rectenna system should be simulated as a whole to validate its feasibility before fabrication. These targeted improvements will advance the development of a fully functional and efficient rectenna system for real-world IoT applications.

## Data Availability

Upon a reasonable request, the datasets obtained and/or analyzed during the current study are available from the corresponding author.

## References

[1] Cansiz, M., Altinel, D. & Kurt, G. K. Efficiency in RF energy harvesting systems: A comprehensive review. *Energy* **174**, 292–309 (2019).

[2] Divakaran, S. K., Krishna, D. D. & Nasimuddin,. RF energy harvesting systems: An overview and design issues. *Int. J. RF Microw. Comput. Aid. Eng.* **29**, e21633 (2019).

[3] Tran, L.-G., Cha, H.-K. & Park, W.-T. RF power harvesting: A review on designing methodologies and applications. *Micro Nano Syst. Lett.* **5**, 14 (2017).

[4] Jameel, M. S., Mezaal, Y. S. & Atilla, D. C. Miniaturized coplanar waveguide-fed UWB antenna for wireless applications. *Symmetry* **15**, 633 (2023).

[5] Karmakar, A. Fractal antennas and arrays: A review and recent developments. *Int. J. Microw. Wirel. Technol.* **13**, 173–197 (2021).

[6] Anguera, J. et al. Fractal antennas: An historical perspective. *Fract. Fract.* **4**, 3 (2020).

[7] Ullah, Md. A. et al. A review on antenna technologies for ambient RF energy harvesting and wireless power transfer: Designs. *Chall. Appl. IEEE Access* **10**, 17231–17267 (2022).

[8] Dahiya, A., Anand, R., Sindhwani, N. & Kumar, D. A novel multi-band high-gain slotted fractal antenna using various substrates for X-band and Ku-band applications. *Mapan* **37**, 175–183 (2022).

[9] Ez-Zaki, F. et al. Double negative (DNG) metamaterial-based koch fractal MIMO antenna design for sub-6-GHz V2X communication. *IEEE Access* **11**, 77620–77635 (2023).

[10] Fakharian, M. M. A wideband fractal planar monopole antenna with a thin slot on radiating stub for radio frequency energy harvesting applications. *Int. J. Eng.* **33**, 2181–2187 (2020).

[11] Parikh, R. & Singh, B. Effects of slots on resonant frequencies of a Microstrip patch antenna. in *2018 Fourth International Conference on Computing Communication Control and Automation (ICCUBEA)* 1–5 (IEEE, Pune, India, 2018). 10.1109/iccubea.2018.8697791.

[12] Donchev, E. et al. The rectenna device: From theory to practice (a review). *MRS Energy Sustain.* **1**, E1 (2014).

[13] Adam, I. et al. Feasibility study on RF energy harvesting in Malaysia. *Adv. Sci. Lett.* **23**, 5034–5038 (2017).

[14] Benhamou, A., Tellache, M., Hebib, S. & Mahfoudi, H. A wide input power range rectenna for energy harvesting and wireless power transfer applications. *Int. J. Microw. Comput. Aid. Eng.* **30**, e22461 (2020).

[15] Eltresy, N., Eisheakh, D., Abdallah, E. & Elhenawy, H. RF energy harvesting using efficiency dual band rectifier. in *2018 Asia-Pacific Microwave Conference (APMC)* 1453–1455 (IEEE, Kyoto, 2018). 10.23919/apmc.2018.8617347.

[16] Nintanavongsa, P., Muncuk, U., Lewis, D. R. & Chowdhury, K. R. Design optimization and implementation for RF energy harvesting circuits. *IEEE J. Emerg. Sel. Top. Circuits Syst.* **2**, 24–33 (2012).

[17] Skyworks Solutions, Inc. Surface Mount Mixer and Detector Schottky Diodes Datasheet. (2007).

[18] Silicon Laboratories Inc. AN1275: Impedance Matching Network Architectures.

[19] Dandgavhal, N. S., Kadu, M. B. & Labade, R. P. Design and simulation of Koch Snowflake fractal antenna for GPS, WiMAX and radar application. in *2015 IEEE Bombay Section Symposium (IBSS)* 1–5 (IEEE, Mumbai, India, 2015). 10.1109/ibss.2015.7456659.

[20] Kharat, K., Dhoot, S. & Vajpai, J. Design of compact multiband fractal antenna for WLAN and WiMAX applications. in *2015 International Conference on Pervasive Computing (ICPC)* 1–4 (IEEE, Pune, India, 2015). 10.1109/pervasive.2015.7087008.

[21] Annou, A., Berhab, S. & Chebbara, F. Koch Snowflake Fractal Patch Antenna with New ENG Metamaterial loads for WiMAX and Wi-Fi. in *2022 7th International Conference on Image and Signal Processing and their Applications (ISPA)* 1–5 (IEEE, Mostaganem, Algeria, 2022). 10.1109/ispa54004.2022.9786294.

[22] Krishna, D. D., Gopikrishna, M., Aanandan, C. K., Mohanan, P. & Vasudevan, K. Compact wideband Koch fractal printed slot antenna. *IET Microw. Anten. Propag.* **3**, 782–789 (2009).

[23] Alex-Amor, A. et al. RF energy harvesting system based on an Archimedean spiral antenna for low-power sensor applications. *Sensors* **19**, 1318 (2019). 30884791 10.3390/s19061318 PMC6471814

[24] Interference coupling mechanisms. in *EMC for Product Designers* 261–297 (Elsevier, 2017). 10.1016/b978-0-08-101016-7.50011-0.

[25] Lu, P., Yang, X.-S., Li, J.-L. & Wang, B.-Z. Polarization reconfigurable broadband rectenna with tunable matching network for microwave power transmission. *IEEE Trans. Anten. Propag.* **64**, 1136–1141 (2016).

[26] Alex-Amor, A., Moreno-Núñez, J., Fernández-González, J. M., Padilla, P. & Esteban, J. Parasitics impact on the performance of rectifier circuits in sensing RF energy harvesting. *Sensors* **19**, 4939 (2019). 31766171 10.3390/s19224939 PMC6891323

[27] Mansour, M. M. & Kanaya, H. Compact RF rectifier circuit for ambient energy harvesting. in *2017 IEEE International Symposium on Radio-Frequency Integration Technology (RFIT)* 220–222 (IEEE, Seoul, South Korea, 2017). 10.1109/rfit.2017.8048256.

[28] Muhammad, S. et al. Broadband RCN-based RF-rectifier with a large range of power for harvesting ambient wireless energy. *AEU: Int. J. Electron. Commun.* **152**, 154228 (2022).

[29] Coskuner, E. & Garcia-Garcia, J. J. Metamaterial impedance matching network for ambient RF-energy harvesting operating at 2.4 GHz and 5 GHz. *Electronics* **10**, 1196 (2021).

[30] Bito, J. et al. A novel solar and electromagnetic energy harvesting system with a 3-D printed package for energy efficient internet-of-things wireless sensors. *IEEE Trans. Microw. Theory Tech.* **65**, 1831–1842 (2017).

[31] Lee, W. et al. Metamaterial-integrated high-gain rectenna for RF sensing and energy harvesting applications. *Sensors* **21**, 6580 (2021). 34640900 10.3390/s21196580 PMC8512327

